# Osteonecrosis of the humeral head in a human immunodeficiency virus-infected patient under tenofovir disoproxil fumarate–emtricitabine–lopinavir/ritonavir for 10 years: a case report

**DOI:** 10.1186/s13256-021-03145-1

**Published:** 2021-12-18

**Authors:** Kalilou Diallo, Bruce Shinga Wembulua, Mohamadou Aidara, Armel Alleyo, Noel Magloire Manga

**Affiliations:** 1 Unit of Infectious and Tropical Diseases, Assane Seck University, Hospital de la Paix, Ziguinchor, Senegal; 2 Department of Infectious and Tropical Diseases, Fann University Hospital, Dakar, Senegal; 3 Unit of Radiology, Hospital de la Paix, Ziguinchor, Senegal; 4 Unit of Orthopedics and Traumatology, Hospital de la Paix, Ziguinchor, Senegal

**Keywords:** Aseptic osteonecrosis, Humeral head, HIV-infected patients, Antiretroviral therapy, Case report

## Abstract

**Background:**

Human immunodeficiency virus-infected patients are 100 times more likely to develop aseptic osteonecrosis compared with the general population. While 90% of cases concern the femoral head, the involvement of humeral bone remains rare.

**Case presentation:**

We report a case of aseptic osteonecrosis of the left humeral head complicating antiretroviral therapy in a female, 46-year-old, Bissau-Guinean human immunodeficiency virus-infected patient received in a context of progressive pain in the left shoulder followed by limitation of articular movements. Standard x-ray of the shoulder allowed us to make the diagnosis by showing a typical image of osteonecrosis. The treatment was medical combined with physiotherapy.

**Conclusions:**

Aseptic osteonecrosis should be systematically looked for in human immunodeficiency virus patients on antiretroviral treatment. In addition to femoral head aseptic necrosis, the involvement of the humeral bone should also be considered.

## Background

Antiretroviral therapy has drastically reduced the morbidity and mortality associated with human immunodeficiency virus (HIV) infection [1]. However, new concerns have arisen from its side effects, some of which affect the bones. HIV-infected patients are 100 times more likely to develop aseptic osteonecrosis (AON) compared with the general population [2]. This concerns in 90% of cases the femoral head [2, 3]. The involvement of the humeral bone remains rare [4]. We report a case of AON of the left humeral head complicating antiretroviral therapy in a patient infected with HIV-2 at the 10th year of treatment.

## Case presentation

**Fig. 1:**
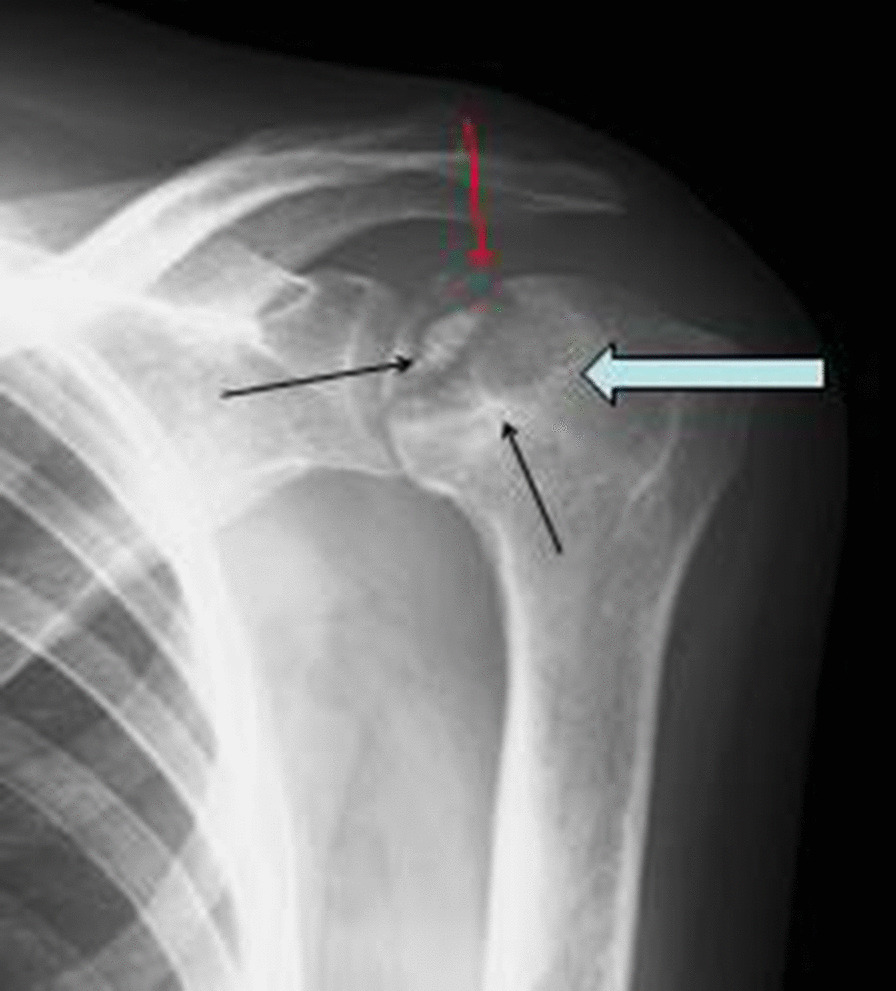
The left shoulder x-ray showed a patch of osteolysis (thick blue arrow) on the humeral head with a clear osteosclerosis border line (small black arrow). The lesion is centered by an osteocondensed image (long black arrow) with an appearance of cortical rupture typical of systemic osteonecrosis (red arrow)

A 46-year-old, Bissau-Guinean patient who had been HIV-2-positive since 2006 was hospitalized in the medical service of the Hospital de la Paix in Ziguinchor, Senegal for pain in the left shoulder of progressive installation, accompanied by limitation of articular movements. These symptoms had been evolving for 2 years before her admission. In her history, there was no indication of trauma, long-term corticosteroid therapy, or diabetes or sickle cell disease. She was under antiretroviral treatment consisting of tenofovir disoproxil fumarate–emtricitabine–lopinavir/ritonavir (TDF-FTC-LPv/r) with good treatment compliance. On general examination, she had clear consciousness, temperature 36.5 °C, respiratory rate (RR) 20 cycles per minute, blood pressure 120/80 mmHg, height 1.72 m, weight 55 kg, body mass index 16.95 kg/m^2^. On physical examination, there was no deformity of the shoulder. However, mobilization was limited by pain. In addition, there was oral candidiasis. Laboratory tests revealed serum creatinine at 25 mg/l (clearance of creatinine 22.06 kg/m^2^), uremia at 0.81 g/l, proteinuria at 2.03 g/24 hours, and serum calcium at 72.4 g/l. Immunovirologically, cluster of differentiation 4 (CD4) count was 24 cells/mm^3^ and the viral load was 55,000 copies/ml. Kidney ultrasound was normal. Left shoulder x-ray showed a patch of osteolysis (Fig. 1, red arrow) on the humeral head with a clear borderline of osteosclerosis (Fig. 1, black arrow). The lesion was centered by an osteocondensed image (Fig. 1, long black arrow) with an appearance of cortical rupture typical of systemic osteonecrosis. We also noted a conservation of the spherical aspect of the humeral head and an integrity of the glenoid of the scapula. This made it possible to classify osteonecrosis as stage III of Ficat and Arlet. The treatment was medical using analgesics and antiinflammatory drugs combined with physiotherapy. This relieved the pain and prevented stiffness of the joint.

## Discussion and conclusions

Bone complications, initially considered rare, are increasingly being reported in patients living with HIV (PLWHIV) [3]. They are 100 times more at risk of developing AON, and this commonly concerns the femoral head [2, 3]. Studies mostly carried out in men over the age of 40 years report the prevalence of osteoporosis in people living with HIV as being between 3% and 22% and that of osteopenia at about 23–65% [5, 6]. Humeral localization remains rare and affects less than 10% of patients with osteonecrosis [7, 8]. The average age of onset is between 40 and 50 years [9, 10]. Our clinical case is one of the rare observations of humeral AON reported in PLWHIV in Senegal.

The diagnosis of osteonecrosis of the humeral head is based on clinical arguments and imaging [magnetic resonance imaging (MRI), bone densitometry, computed tomography (CT), or x-ray]. The pain with limitation of joint movements observed in our patient is a classic revealing sign, although a few asymptomatic cases are also reported [2]. The standard x-ray of the shoulder allowed us to make the diagnosis by showing a typical image of osteonecrosis.

The etiology of osteonecrosis during HIV infection is multifactorial. The initiation of antiretroviral therapy (ART) leads to a reduction in bone mineral density [2] comparable in magnitude to that observed during the perimenopausal period and during corticosteroid therapy [8]. This bone loss is greater on tenofovir disoproxil fumarate (TDF) regimens, less with integrase inhibitors, and rarely with protease inhibitors (PI) [11]. One year on a TDF-based regimen multiplies the risk of osteonecrosis by 4 [12].

Corticosteroid use and alcoholism are also essential risk factors for AON but not specific for HIV [2, 12]. Anticardiolipid antibodies, common in patients with HIV infection, have also been implicated as they promote damage to the vascular endothelium, platelet aggregation, and vascular thrombosis. The acquired protein S deficiency would be an associated factor [13]. History of acquired immunodeficiency syndrome (AIDS) and duration of infection, low CD4 count, and duration of exposure to combination antiretroviral drugs are other factors described in the literature [2, 14]. Our patient had been on TDF/FTC/LPv/r for 10 years with a low CD4 count. She was not an alcoholic or diabetic and was not on a corticosteroid. In addition, renal failure with hypocalcemia observed in our patient may have played a role in bone weakening.

The pathophysiology of osteonecrosis is poorly understood, but the main mechanism is vascular occlusion leading to bone hypoxia and necrosis [13, 14]. Inflammation, as well as bleeding disorders demonstrated by high levels of D-dimer and C-reactive protein, was also linked to the risk of osteonecrosis [15].

The management of AON in HIV-infected patients has no specificity compared with the general population [16]. At the early stage, treatment is medical, combining analgesics with physiotherapy. Drilling with bone marrow transplants can also be proposed. Later, the treatment is surgical and consists of cementoplasty, removal of sequesters, or total shoulder prosthesis when there is glenohumeral arthritis [13, 17–19].

Antiretroviral treatments are responsible for severe complications that require regular monitoring. Aseptic osteonecrosis associated with TDF or protease inhibitors should be systematically looked for to prevent the progression to terminal stages. In addition to the femoral head, the involvement of the humeral bone should also be considered.

## Data Availability

All relevant data and materials are included in the manuscript.

## Abbreviations

AON: Aseptic osteonecrosis
IP: Protease inhibitors
ART: Antiretroviral therapy
PLWHIV: Patients living with HIV
TDF: Tenofovir disoproxil fumarate
LPV/r: Lopinavir/ritonavir
FTC: Emtricitabine
